# Supplementary material for: Bacteria Optimize Tumble Bias to Strategically Navigate Surface Constraints
Source: Adv Sci (Weinh). 2025 Jun 20;12(34):e02063. doi: 10.1002/advs.202502063 (PMC12442700; doi:10.1002/advs.202502063)
Supplement: Supplementary file 1 — Supporting Information [file ADVS-12-e02063-s001.docx]

**Supporting Information for**

**“Bacteria optimize tumble bias to strategically navigate surface constraints”**

Antai Tao^1^, Guangzhe Liu^1,2,3^, Rongjing Zhang^1,*^, Junhua Yuan^1,*^

^1^ Hefei National Research Center for Physical Sciences at the Microscale and Department of Physics, University of Science and Technology of China, Hefei, Anhui 230026, China.

^2^ Wenzhou Institute, University of Chinese Academy of Science, Wenzhou, Zhejiang 325000, P.R. China

^3^ School of Engineering and science, University of Chinese Academy of Science, Beijing 100049, P.R. China

* Corresponding authors: Junhua Yuan or Rongjing Zhang.

Email address: jhyuan@ustc.edu.cn (J. Yuan), rjzhang@ustc.edu.cn (R. Zhang).

**This PDF file** **includes:**

Notes S1 to S4

Table S1

Illustration of Movies S1 to S3

Figures S1 to S8

**Supplementary Notes**

**Note S1. Estimation of the mean tumble bias of wild-type *E. coli* using the mean CW bias of single flagellum**

In a previous study, Mears *et al.* found that the swimming behaviors of *E. coli cells* are robust against variations in the number of flagella due to inter-flagellar correlations^1^. In their model, TB of a wild-type cell can be computed as:

$$\mathrm{TB}=1-\left( 1-\mathrm{CB} \right)^{N_{\mathrm{eff}}} , (S5)$$

where the CW bias (CB) denotes the fraction of time during which a single flagellar motor spins clockwise, and *N*_eff_ denotes the effective number of flagella, which can be approximated by:

$$N_{\mathrm{eff}}=1.27\times N_{\mathrm{flag}}^{0.5} , (S6)$$

where *N*_flag_ denotes the actual flagellar number. According to the observation by Turner *et al.*, the mean value of *N*_flag_ is ~ 3 for wild-type *E. coli* (AW405)^2^. The CW bias for single flagellum was measured to show a peak distribution with a mean value of ~ 0.12^1,3^. Consequently, the mean tumble bias of wild-type *E. coli* cells is estimated to be 0.25 by Equation (S6), which agrees well with our measurements (0.24 ± 0.13, Figure 3f in the main text) within experimental error.

**Note S2: Three-dimensional tracking near the surface**

*E. coli* cells are rod-shaped, varying in length (end to end) but having nearly identical width (diameter of hemispherical end caps), approximately 1 μm wide^4^. This uniform width allows for the reconstruction of the *z* coordinate using the principle of Rayleigh-Sommerfeld back-scattering^5,6^. For those biological specimens with refractive index close to that of their surroundings, they act as phase objects with negligible absorption, rendering them almost invisible when they are located in the focal plane of a bright-field imaging system. However, a small amount of defocusing makes them visible again by producing a blurred image. Specifically, when an object initially positioned in the focal plane moves away from the objective, the central part of the cast image appears bright with a dark halo. Conversely, if the object moves closer to the objective, the central part of the cast image appears dark with a bright halo (Figure S1). The halo width depends on the displacement of the object from the focal plane^7^. In the present study, considering that *E. coli* cells have a similar width, we define the halo width as the displacement between two intensity peaks or valleys in the pixel intensity cross-section along the line perpendicular to the major axis of the cell (Figure S1).

For the calibration of the relationship between the halo width and the displacement away from the focal plane (Δ*z*), *E. coli* cells were fixed to the coverslip surface coated with poly-L-lysine (0.01%, Sigma). Δ*z* was adjusted within the range of −8 to 8 μm using a piezo sample stage, and a bright-field image was recorded at every 1 μm interval. As shown in Figure S2a, the mean halo width versus Δ*z* exhibits a nearly axisymmetric linear relationship for Δ*z* values within the range of −8 to −1 μm and from 1 to 8 μm. Therefore, Δ*z* can be determined based on both the color (dark or bright) and the width of halo. To assess the accuracy of the tracking method, for *x* and *y* direction, localization errors were determined as the standard deviation of the lateral position. For *z* direction, localization errors are determined as the residuals of the extracted *z* position against the known *z* position. As the magnitude of these errors depends on the *z* position, we compute the root mean square error (RMSE) across 20 different bacteria located at various *z* position. As shown in Figure S2b, we conclude that the localization errors are less than 0.8 μm. According to the analysis by Junot *et al.*^8^, the bacterial mean surface residence time should not be strongly affected by the sub-micron-scale localization errors.

Note that the cell body is not always parallel to the focal plane during the near-surface motion. Hence, it is important to verify whether the halo width can be effectively used to determine Δ*z* for those tilted cells. To address this, we trapped a cell and adjusted the *z* position to the same value for the two separate optical tweezers Figure S2c. A bright-field image was recorded to calculate the reference displacement Δ*z*_0_ using the halo width. Subsequently, we moved *z* position of trap #1 upwards and that of trap #2 downwards by the same displacement *z_j_* and calculated Δ*z_j_* in the same manner. The location error of individual tilted cells was calculated as Δ*z_j_* - Δ*z*_0_. This analysis was conducted for dozens of bacteria with varying obliquities, and the location errors for Δ*z* > 0 and Δ*z* < 0 were found to be 0.07 ± 0.42 μm and −0.34 ± 0.59 μm (Mean ± SD), respectively. Therefore, the halo width can be effectively utilized to estimate Δ*z* within the range of −8 to 8 μm.

To determine the absolute *z* position of the cell at the surface (*z*_cell_ = 0), we randomly selected a bacterium adhered and parallel to the surface, or observed a time period when a bacterium was swimming smoothly in clockwise circular motion within the visual field^9^. We analyzed the minimum Δ*z* (denoted as Δ*z*_s_) from the bright-field images during these instances. Subsequently, for each frame, Δ*z* could be transformed into *z*_cell_ as: *z*_cell_ = Δ*z* − Δ*z*_s_. This method allowed us to reconstruct the three-dimensional bacterial trajectory near the surface. Note that in cases where −1 < Δ*z* < 1 μm, the bright-field image of the bacterium became too blurred to be distinguished, and *z*_cell_ in these frames was approximated using linear interpolation. As an example, Movie S1 shows a reconstructed three-dimensional bacterial trajectory. Additionally, during the data acquisition process, the sample stage was manually adjusted in the *xy*-plane when necessary to keep the target cell within view throughout the recording. The shift of the sample stage in the *xy*-plane can be corrected by measuring the movement of immobile impurities in the visual field (Movie S2), although *z*_cell_ would not be affected by the shift in the *xy*-plane.

**Note S3: Simulation of bacterial trajectories near the surface**

The flow chart of the simulation is shown in Figure 3c in the main text. Initially, Tumble bias (TB) was randomly generated in the range of 0 to 1, and the cell’s initial position $\vec{r}(t=0)$ was set to (0, 0, 9) (unit: μm) to simulate a surface arrival-escape process. The initial state of bacterium was determined by generating another random number *A* in the range of 0 to 1 and comparing the values of *A* and TB. The initial orientation $\hat{e}(t=0)$ was determined by randomly generated polar angle *φ* in [0, π] and azimuth angle *θ* in [0, 2π). The mean swimming velocity *V* was set to 19 μm/s (Figure 2e in the main text). *D*_t_ was set to 6.6 μm^2^/s (Figure 2b in the main text). *D*_r_ and *D*_θ_ were respectively set to 0.025 s^-1^ and 3.5 s^-1^ according to previously measured or fitted results^10-12^. Considering the phenotypic variation of *E. coli* cells^9^, three different values (15/25/35 μm) were applied to the radius (*R*) of curvature of surface circular trajectories. Run and tumble intervals were simulated as Poisson processes with transition rates *k*_RT_ and *k*_TR_, which could be computed using Equation (3) in the main text. The timestep (Δ*t*) of simulation was set in consideration of the associated diffusion time ($\tau$) of rotational diffusion, which is defined as:

$$\tau=\frac{1}{2D_{s}}, (S1)$$

where *D*_s_ is the rotational diffusion coefficient. At short times ($\Delta t\leq\tau$), the bacterium keeps the memory of its orientation. At longer times ($\Delta t\gg\tau$), it loses directional memory, transitioning from anisotropic to isotropic diffusion. Therefore, it is important to set the simulation timestep shorter than $\tau$ for continuous stochastic processes. In our model, the rotational diffusion coefficients for *E. coli* cells in run and tumble states are *D*_r_ = 0.025 s^-1^ and *D*_θ_ = 3.5 s^-1^, respectively. Consequently, for run and tumble states, $\tau$ can be computed to be 40 s and 0.14 s, respectively. To implement the simulations with enough accuracy while controlling the consumption of computing resources, we set the timestep to 0.01 s, one order of magnitude shorter than the minimal $\tau$ (0.14 s).

For each timestep, the changes of the cell’s centroid position $\vec{r}=(x,y,z)$ and orientation $\hat{e}$ with polar angle *φ* and azimuth angle *θ* (Figure 3b in the main text), can be described by integrating the stochastic differential equations (SDEs) of spatial motion [Equations. (4-6) in the main text] using the Euler-Maruyama method. When the running bacterium moves away from the surface (i.e. *z* > 0), iterations follow:

$$\vec{r}\left( t+\Delta t \right)=\vec{r}\left( t \right)+V\hat{e}\left( t \right)\Delta t,$$

$$\hat{e}\left( t+\Delta t \right)=\hat{e}\left( t \right)+\sqrt{2D_{r}\Delta t}\eta\left( t \right) , (S2)$$

where $\eta(t)$ is a Gaussian white noise of unit variance with$\left\langle\eta(t_{1})\eta(t_{2}) \right\rangle=\delta(t_{1}-t_{2})$. When the running bacterium moves along the surface (i.e. *z* = 0), iterations follow:

$$\vec{r}\left( t+\Delta t \right)=\vec{r}\left( t \right)+V\hat{e}\left( t \right)\Delta t,$$

$$\theta\left( t+\Delta t \right)=\theta\left( t \right)+\Omega\Delta t+\sqrt{2D_{r}\Delta t}\eta\left( t \right),$$

$$\varphi\left( t+\Delta t \right)=\varphi\left( t \right)\equiv\frac{\pi}{2}. (S3)$$

For the tumbling bacterium, iterations follow:

$$x\left( t+\Delta t \right)=x\left( t \right)+\sqrt{2D_{t}\Delta t}\xi_{1}\left( t \right),$$

$$y\left( t+\Delta t \right)=y\left( t \right)+\sqrt{2D_{t}\Delta t}\xi_{2}\left( t \right),$$

$$z\left( t+\Delta t \right)=z\left( t \right)+\sqrt{2D_{t}\Delta t}\xi_{3}\left( t \right),$$

$$\hat{e}\left( t+\Delta t \right)=\hat{e}\left( t \right)+\sqrt{2D_{\theta}\Delta t}\eta\left( t \right), (S4)$$

where $\xi_{1}\left( t \right)$, $\xi_{2}\left( t \right)$, $\xi_{3}\left( t \right)$ and $\eta(t)$ are independent Gaussian white noises of unit variance. Considering the steric hindrance of the surface, the displacement in the *z*-direction will be further adjusted as: if $z\left( t+\Delta t \right)<0$, we set $z\left( t+\Delta t \right)=0$. After a time period containing a run and a tumble, if $z$> 20 μm, the simulation was terminated, and the generated track was used for analysing *T*_s_ if there existed the surface arrival-escape process (*z* > 8 μm → *z* < 3 μm → *z* > 8 μm). The parameter values used in the simulation were summarized in Table S1.

**Note S4. Theoretical computation of the effective two-dimensional diffusion coefficient**

As described in the main text, we only consider the bacterial motion in the *xy*-plane, consequently, the probability density of finding the bacterium in state R (run) or T (tumble) at position$\vec{r}=(x,y)$with azimuth angle$\theta$at time$t$, denoted as$p_{k=R,T}(\vec{r},\theta,t)$, can be obtained from the spatiotemporal evolution described by the following Fokker-Planck equations with state transition jumps:

$$\frac{\partial p_{R}(\vec{r},\theta,t)}{\partial t}=-V\hat{e}\left( t \right)\cdot\nabla p_{R}(\vec{r},\theta,t)-\Omega\frac{\partial p_{R}(\vec{r},\theta,t)}{\partial\theta}+D_{r}\frac{\partial^{2}p_{R}(\vec{r},\theta,t)}{\partial\theta^{2}}$$

$$-k_{\mathrm{RT}}p_{R}(\vec{r},\theta,t)+k_{\mathrm{TR}}p_{T}(\vec{r},\theta,t),$$

$$\frac{\partial p_{T}(\vec{r},\theta,t)}{\partial t}=D_{t}\nabla^{2}p_{T}(\vec{r},\theta,t)+D_{\theta}\frac{\partial^{2}p_{T}\left( \vec{r},\theta,t \right)}{\partial\theta^{2}}-k_{\mathrm{TR}}p_{T}(\vec{r},\theta,t)+k_{\mathrm{RT}}p_{R}(\vec{r},\theta,t). (S7)$$

To solve the equations, we followed a similar method described previously^13^. We used the Taylor-Kubo formula for simplification^14^:

$$\mathrm{MSD}_{xy}(t)= \left\langle\left| \vec{r}\left( t \right)-\vec{r}\left( 0 \right) \right|^{2} \right\rangle=2\int_{0}^{t} dt^{'}\int_{0}^{t^{'}} dt^{''}\left\langle\vec{v}\left( t^{'} \right)\cdot\vec{v}\left( t^{''} \right) \right\rangle. (S8)$$

We first integrated$p_{R,T}(\vec{r},\theta,t)$over space:

$$\tilde{p}_{R,T}(\theta,t)=\int d\vec{r}p_{R,T}\left( \vec{r},\theta,t \right). (S9)$$

Then, Equation (S7) could be rewritten as:

$$\frac{\partial\tilde{p}_{R}(\theta,t)}{\partial t}=-\Omega\frac{\partial\tilde{p}_{R}\left( \theta,t \right)}{\partial\theta}+D_{r}\frac{\partial^{2}\tilde{p}_{R}\left( \theta,t \right)}{\partial\theta^{2}}-{k_{\mathrm{RT}}\tilde{p}}_{R}(\theta,t)+k_{\mathrm{TR}}\tilde{p}_{T}(\theta,t),$$

$$\frac{\partial\tilde{p}_{T}(\theta,t)}{\partial t}=D_{\theta}\frac{\partial^{2}\tilde{p}_{T}(\theta,t)}{\partial\theta^{2}}-k_{\mathrm{TR}}\tilde{p}_{T}(\theta,t)+k_{\mathrm{RT}}\tilde{p}_{R}(\theta,t). (S10)$$

By performing a Fourier series expansion of$\tilde{p}_{R,T}(\theta,t)$: $\tilde{p}_{R,T}(\theta,t)=\sum_{n=-\infty}^{+\infty} \hat{p}_{R,T}(n,t)e^{in\theta}$, we obtained a system of linear differential equations:

$$\left[ \begin{matrix} \frac{\partial\hat{p}_{R}(n,t)}{\partial t} \\ \frac{\partial\hat{p}_{T}(n,t)}{\partial t} \end{matrix} \right]=\left[ \begin{matrix} -(in\Omega+D_{r}n^{2}+k_{\mathrm{RT}}) & k_{\mathrm{TR}} \\ k_{\mathrm{RT}} & -(D_{\theta}n^{2}+k_{\mathrm{TR}}) \end{matrix} \right]\left[ \begin{matrix} \hat{p}_{R}(n,t) \\ \hat{p}_{T}(n,t) \end{matrix} \right], (S11)$$

where$\hat{p}_{R,T}(n,t)=\frac{1}{2\pi}\int_{-\pi}^{\pi} d\theta\tilde{p}_{R,T}(\theta,t)e^{-in\theta}$. The solution of Equation (S11) can be written as:

$$\left[ \begin{matrix} \hat{p}_{R}(n,t) \\ \hat{p}_{T}(n,t) \end{matrix} \right]= \sum_{s=1}^{2} C_{s}(n)X_{s}(n)e^{\lambda_{s}(n)t}, (S12)$$

where *λ*_s_ and *X*_s_ are eigenvalues and eigenvectors of the matrix in Equation (S11), and *C*_s_ can be calculated with the initial condition:

$$\left[ \begin{matrix} C_{1}(n) \\ C_{2}(n) \end{matrix} \right]=X^{-1}\left[ \begin{matrix} \hat{p}_{R}(n,0) \\ \hat{p}_{T}(n,0) \end{matrix} \right], (S13)$$

where *X*^-1^ is the inverse of *X­­*, which is the matrix composed of all eigenvectors. Finally, we obtained:

$$\tilde{p}_{k}(\theta,t)=\sum_{n=-\infty}^{+\infty} \hat{p}_{k}\left( n,t \right)e^{in\theta}=\sum_{n=-\infty}^{+\infty} \sum_{s=1}^{2} C_{s}\left( n \right)X_{\mathrm{ks}}\left( n \right)e^{\lambda_{s}\left( n \right)t+in\theta}, (S14)$$

where k = 1 and 2 correspond to run (R) and tumble (T) states, respectively.

Next, we considered the velocity correlation in Equation (S8) during run periods, which can be written with the following expansion:

$$\left\langle\vec{v}\left( t' \right)\cdot\vec{v}(t'') \right\rangle=\int d\theta'\int d\theta''V^{2}cos(\theta'-\theta'')\tilde{p}(R,\theta',t'\cap R,\theta'',t'')$$

$$=\int d\theta^{'}\int d\theta^{''}V^{2}cos(\theta'-\theta'')\tilde{p}(R,\theta',t'|R,\theta'',t'')\tilde{p}(R,\theta'',t'') , (S15)$$

where $\tilde{p}(R,\theta',t'\cap R,\theta'',t'')$ is the joint probability that a bacterium in the run state with orientation *θ′* at time *t′* and in the run state with orientation *θ″* at time *t″*. Due to time and space homogeneity, the conditional probability can be written as:

$$\tilde{p}(R,\theta',t'|R,\theta'',t'')=\tilde{p}(R,\theta'-\theta'',t'-t''|R,0, 0) , (S16)$$

which could be solved by Equation (S10) with the initial condition$\tilde{p}(R,\theta, 0)=\delta(\theta)$, that is, $\hat{p}_{R}(n,0)=1/2\pi$and$\hat{p}_{T}(n,0)=0$. Therefore,$C_{s}(n)={{(X}^{-1})}_{s1}/2\pi$. Due to the orthogonality of trigonometric function, Equation (S15) can be rewritten as:

$$\left\langle\vec{v}\left( t' \right)\cdot\vec{v}(t'') \right\rangle=V^{2}\sum_{n=-\infty}^{+\infty} \sum_{s=1}^{2} C_{s}(n)X_{1s}(n)e^{\lambda_{s}(n)(t^{'}-t^{''})}\times$$

$$\int_{-\pi}^{\pi} d\theta''p(R,\theta'',t'')\int_{-\pi-\theta^{''}}^{\pi-\theta^{''}} d(\theta'-\theta'')cos(\theta'-\theta'')e^{in(\theta^{'}-\theta^{''})}$$

$$=V^{2}(1-\mathrm{TB})\pi\sum_{s=1}^{2} [C_{s}(1)X_{1s}(1)e^{\lambda_{s}(1)(t^{'}-t^{''})}+C_{s}(-1)X_{1s}(-1)e^{\lambda_{s}(-1)(t^{'}-t^{''})}]$$

$$=V^{2}(1-\mathrm{TB})\pi\sum_{s=1}^{2} [C_{s}(1)X_{1s}(1)e^{\lambda_{s}\left( 1 \right)\left( t^{'}-t^{''} \right)}+\bar{C}_{s}(1)\bar{X}_{1s}(1)e^{\bar{\lambda}_{s}\left( 1 \right)\left( t^{'}-t^{''} \right)}] , (S17)$$

where$\bar{C}_{s}$,$\bar{X}_{1s}$and$\bar{\lambda}_{s}$denote the conjugate of *C*_s_, *X*_1s_ and *λ*_s_, respectively. After integrating the velocity correlation over time from Equation (S8), and considering the contribution of Brownian motion during tumble periods, the final MSD can be written as:

$$\left\langle\left| \vec{r}\left( t \right)-\vec{r}\left( 0 \right) \right|^{2} \right\rangle=4TBD_{t}t+2\pi(1-\mathrm{TB})V^{2}\times$$

$$\sum_{s=1}^{2} [C_{s}(1)X_{1s}(1)(\frac{e^{\lambda_{s}(1)t}-1}{{\lambda_{s}(1)}^{2}}-\frac{t}{\lambda_{s}(1)})+\bar{C}_{s}(1)\bar{X}_{1s}(1)(\frac{e^{\bar{\lambda}_{s}\left( 1 \right)t}-1}{{\bar{\lambda}_{s}\left( 1 \right)}^{2}}-\frac{t}{\bar{\lambda}_{s}\left( 1 \right)})] . (S18)$$

With the above analysis, the effective two-dimensional diffusion coefficient (*D_xy_*) can be computed as:

$$D_{xy}=\lim_{t\to\infty} \frac{\left\langle\left| \vec{r}\left( t \right)-\vec{r}\left( 0 \right) \right|^{2} \right\rangle}{4t}=\mathrm{TB}D_{t}+\frac{\pi}{2}V^{2}(1-\mathrm{TB})\sum_{s=1}^{2} -\left( \frac{C_{s}\left( 1 \right)X_{1s}\left( 1 \right)}{\lambda_{s}\left( 1 \right)}+\frac{\bar{C}_{s}\left( 1 \right)\bar{X}_{1s}\left( 1 \right)}{\bar{\lambda}_{s}\left( 1 \right)} \right). (S19)$$

Finally, after calculating *λ*_s_, *X*_s_ and *C*_s_, we obtained:

$$D_{xy}=\mathrm{TB}D_{t}+\frac{V^{2}}{2}\left( 1-\mathrm{TB} \right)\frac{\left( D_{\theta}+k_{\mathrm{TR}} \right)\left( D_{r}k_{\mathrm{TR}}+D_{\theta}k_{\mathrm{RT}}+D_{r}D_{\theta} \right)}{\left( D_{\theta}+k_{\mathrm{TR}} \right)^{2}\frac{V^{2}}{R^{2}}+\left( D_{r}k_{\mathrm{TR}}+D_{\theta}k_{\mathrm{RT}}+D_{r}D_{\theta} \right)^{2}} . (S20)$$

**Supplementary References**

1 Mears, P. J., Koirala, S., Rao, C. V., Golding, I. & Chemla, Y. R. *Escherichia coli* swimming is robust against variations in flagellar number. *eLife* **3**, e01916 (2014).

2 Turner, L., Ryu, W. S. & Berg, H. C. Real-time imaging of fluorescent flagellar filaments. *J. Bacteriol.* **182**, 2793–2801 (2000).

3 Liu, G., Tao, A., Zhang, R. & Yuan, J. Robustness in an ultrasensitive motor. *mBio* **11**, e03050-03019 (2020).

4 Berg, H. C. *E. coli in motion*. (Springer, 2004).

5 Wilson, D. & Zhang, R. 3D localization of weak scatterers in digital holographic microscopy using Rayleigh-Sommerfeld back-propagation. *Opt. Express* **20**, 16735–16744 (2012).

6 Wilson, L. G., Carter, L. M. & Reece, S. E. High-speed holographic microscopy of malaria parasites reveals ambidextrous flagellar waveforms. *Proc. Natl. Acad. Sci. U. S. A.* **110**, 18769–18774 (2013).

7 Bukatin, A., Kukhtevich, I., Stoop, N., Dunkel, J. & Kantsler, V. Bimodal rheotactic behavior reflects flagellar beat asymmetry in human sperm cells. *Proc. Natl. Acad. Sci. U. S. A.* **112**, 15904–15909 (2015).

8 Junot, G. *et al.* Run-to-tumble variability controls the surface residence times of *E. coli* bacteria. *Phys. Rev. Lett.* **128**, 248101 (2022).

9 Lauga, E., DiLuzio, W. R., Whitesides, G. M. & Stone, H. A. Swimming in circles: motion of bacteria near solid boundaries. *Biophys. J.* **90**, 400–412 (2006).

10 Berg, H. C. & Brown, D. A. Chemotaxis in *Escherichia coli* analysed by three dimensional tracking. *Nature* **239**, 500–504 (1972).

11 Saragosti, J., Silberzan, P. & Buguin, A. Modeling *E. coli* tumbles by rotational diffusion. Implications for chemotaxis. *PLoS ONE* **7**, e35412 (2012).

12 Figueroa-Morales, N. *et al.* 3D spatial exploration by *E. coli* echoes motor temporal variability. *Phys. Rev. X* **10**, 021004 (2020).

13 Ipiña, E. P., Otte, S., Pontier-Bres, R., Czerucka, D. & Peruani, F. Bacteria display optimal transport near surfaces. *Nat. Phys.* **15**, 610–615 (2019).

14 Kubo, R. Statistical-mechanical theory of irreversible processes. I. General theory and simple applications to magnetic and conduction problems. *J. Phys. Soc. Jpn.* **12**, 570-586 (1957).

15 Taute, K. M., Gude, S., Tans, S. J. & Shimizu, T. S. High-throughput 3D tracking of bacteria on a standard phase contrast microscope. *Nat. Commun.* **6**, 8776 (2015).

**Supplementary Tables**

**Table S1.** Parameter values for the simulation.

**Parameters Setting values Source or Reference**

Δ*t* 0.01 s ——

*V* 19 μm/s This work

*D*_r_ 0.025 s^-1^  Ref ^12^

*R* 15/25/35 μm Ref ^9^

*D*_t_ 6.6 μm^2^/s This work

*D*_θ_ 3.5 s^-1^ Ref ^10,11^

TB Randomly generated in (0, 1) ——

SF Derived from TB (Equation (2) in the main text) This work

*k*_RT_ Derived from TB and SF (Equation (3) in the main text) This work

*k*_TR_ Derived from TB and SF (Equation (3) in the main text) This work

**Supplementary Movies**

**Movie S1.** A typical high-frame-rate video showing an *E. coli* cell with its poles captured by dual optical traps. The centers of dual optical traps are marked by red crosses. The cell body displayed oscillatory wobble and erratic motion during run and tumble periods, respectively.

**Movie S2.** Reconstruction of three-dimensional bacterial trajectories near the surface. Unwanted background features have been removed using the method described elsewhere^15^. The red segment of the trajectory curve represents the surface residence time period.

**Movie S3.** Reconstruction of three-dimensional bacterial trajectories near the surface with correction for the shift of sample stage in the *xy*-plane. The red segment of the trajectory curve represents the surface residence time period.

**Supplementary Figures**

**Figure S1.** Distribution of cell lengths for individual *T*_s_ samples in our statistical analysis.


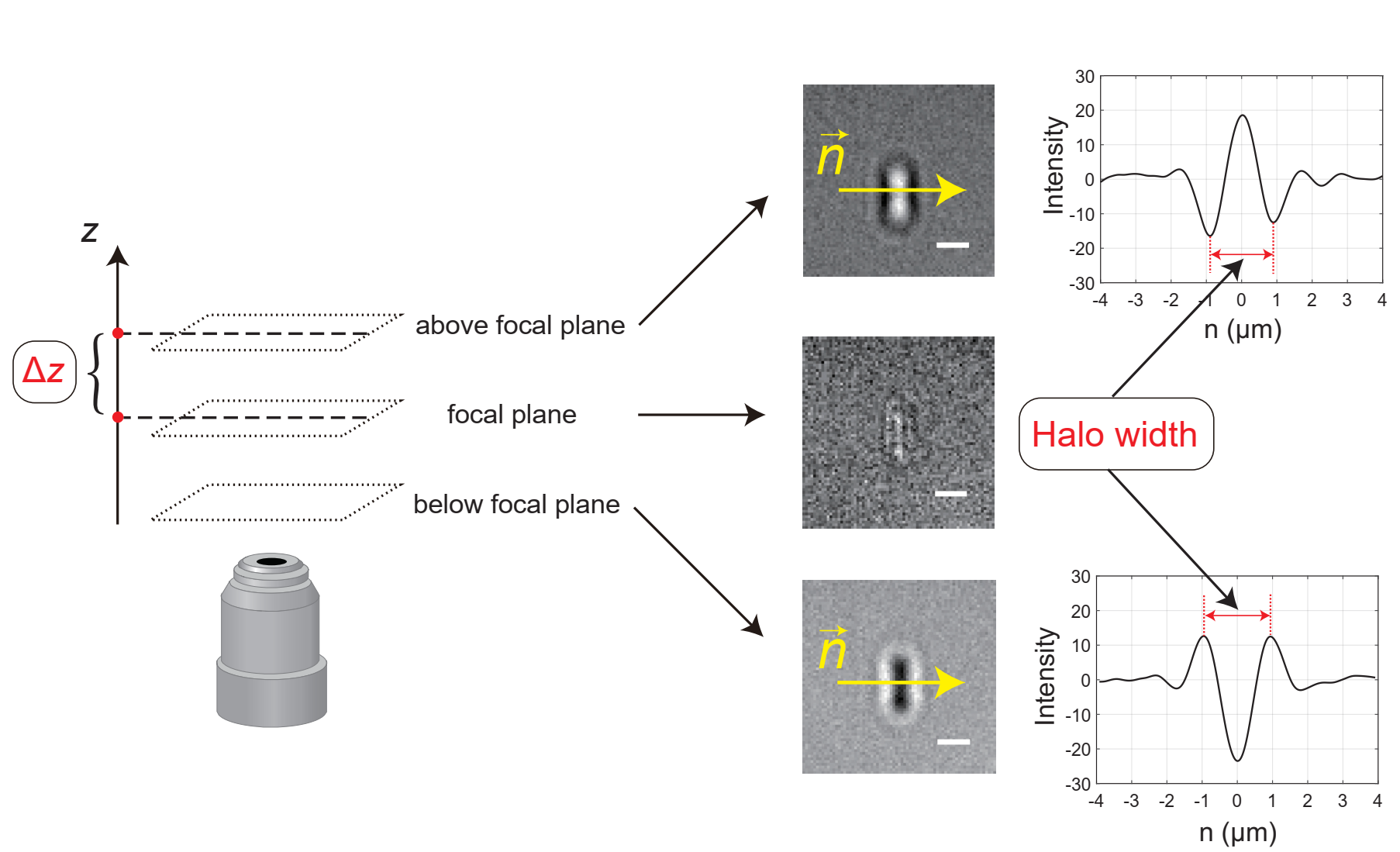


**Figure S2.** Bright-field imaging of *E. coli* cells. In the left panel, Δ*z* denotes the displacement away from the focal plane. Bacteria are nearly invisible when located in the focal plane. However, a small amount of defocusing makes them visible again by casting a blurred image with a surrounding halo. The image contrast has been adjusted for clear presentation (Scale bar: 2 μm). The vector $\vec{n}$, with a length of 8 μm, denotes the line segment normal to the major axis of the cell, centered at the cell centroid. The pixel intensities (after subtracting the mean background) cross section along $\vec{n}$ are shown in the right panel. The halo width is defined as the displacement between two intensity peaks or valleys in the halo.


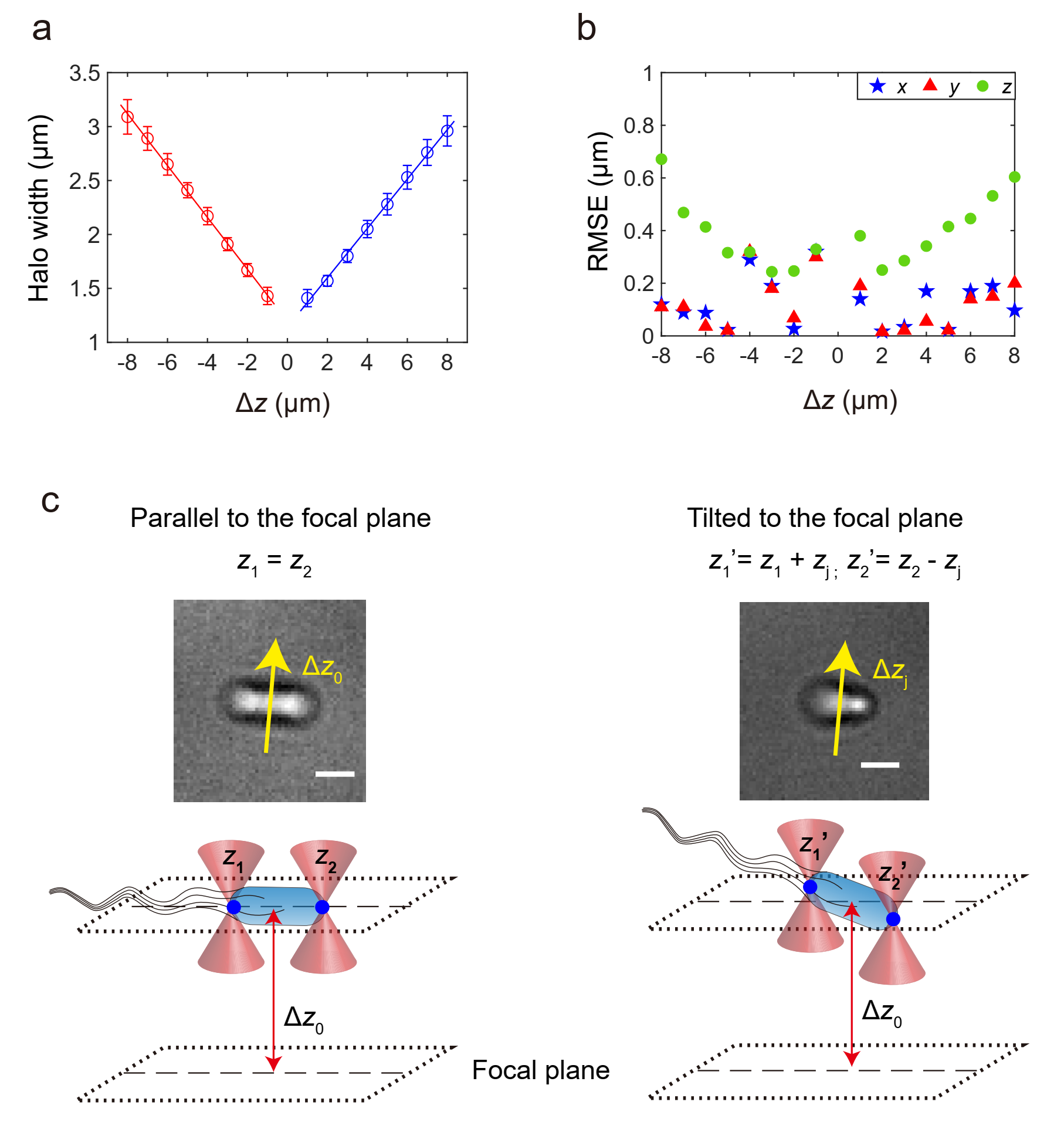


**Figure S3.** Localization accuracy of the tracking method. a) Halo width versus the displacement away from the focal plane (Δ*z*). Error bars denote standard deviation. The red and blue lines represent linear fitting for for Δ*z* in the range of [-8, -1] and [1, 8] (μm), respectively. b) Root mean square error (RMSE) of localization in *x*, *y* and *z*-direction at various *z* position. Errors are determined by analying 20 bacteria immobilized in the sample. c) Illustration for calculating the location error of tilted cells (Scale bar: 2 μm). Firstly, *z* positions of two separate optical tweezers (denoted as *z*1 and *z*2) are adjusted to the same value to analyze the reference displacement Δ*z*_0_ using the halo width. Then, *z*1 and *z*2 are moved the same distance *z_j_* in the opposite direction, and the displacement Δ*z_j_* is analyzed to calculate the location error Δ*z_j_* - Δ*z*_0_.

**Figure S4.** Quantification of temperature variation induced by the optical traps. a) Normalized fluorescence of BCECF solution in relation to temperature. The red dotted line represents the result of linear fitting. Error bars denote standard deviation of three independent measurements. b) Illustration of three regions of interest at different distances from the optical traps. The centers of the dual optical traps are marked by red crosses. Region 1 is a 55 × 55 μm square area centered between the dual optical traps. Region 2 is a hollow square area within a 110 × 110 μm square, excluding Region 1. Region 3 is a hollow square area within a 165 × 165 μm square, excluding Regions 1 and 2. c) Variation of the average temperature in the three regions shown in (b). The laser of the optical traps was turned on at 60 s and turned off at 240 s (corresponding to a trapping time of 180 s in our data collection).

**Figure S5.** Simulated surface residence time in relation to tumble bias. Each graph represents the statistical results of 100000 individual simulated bacterial surface arrival-escape trajectories. Error bars denote SEM.


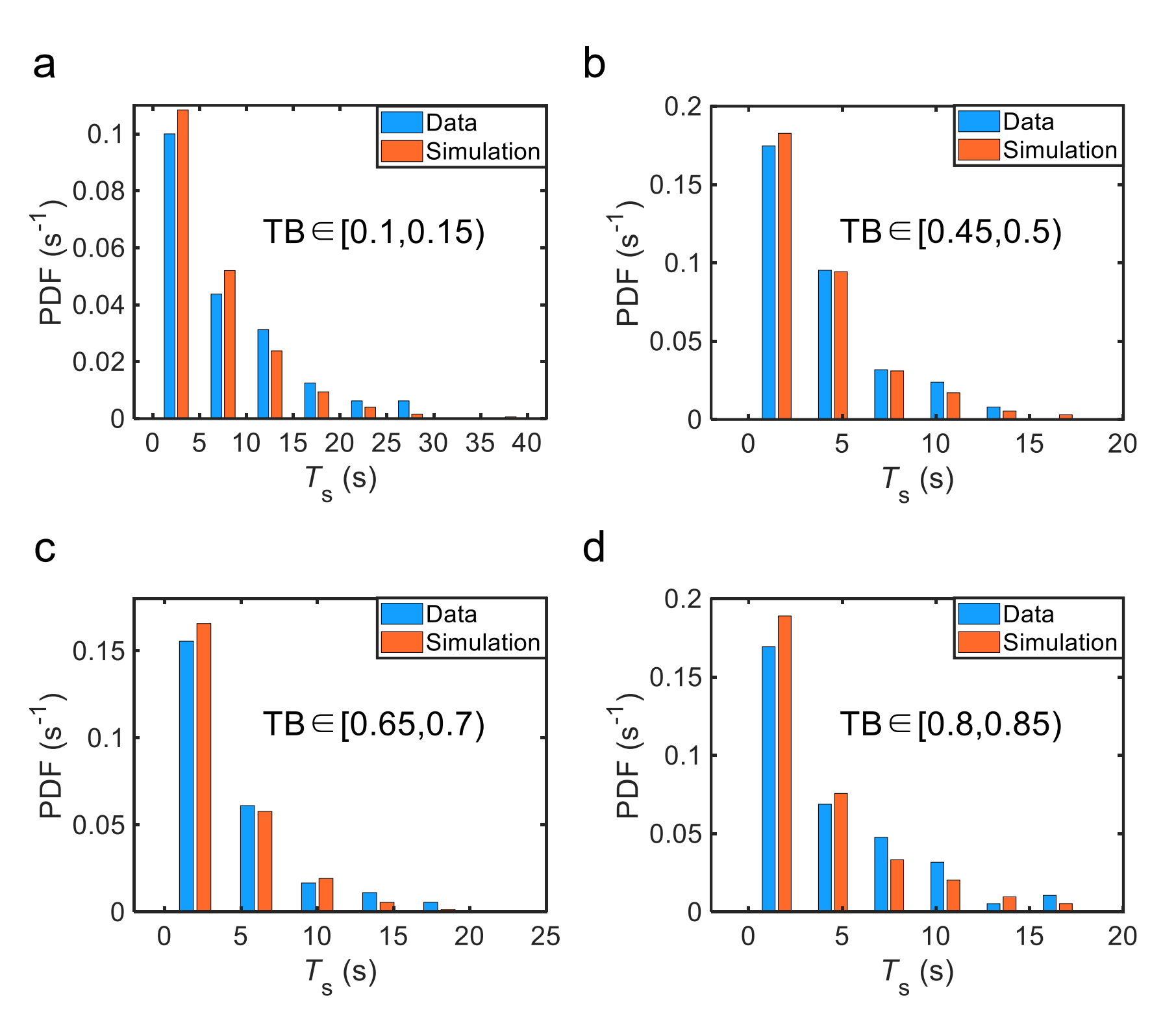


**Figure S6.** Comparison of experimental and simulated distributions of the bacterial surface residence time (*T*_s_) after classification by TB. Results in four different bins of TB (see inset text) are shown here as examples.


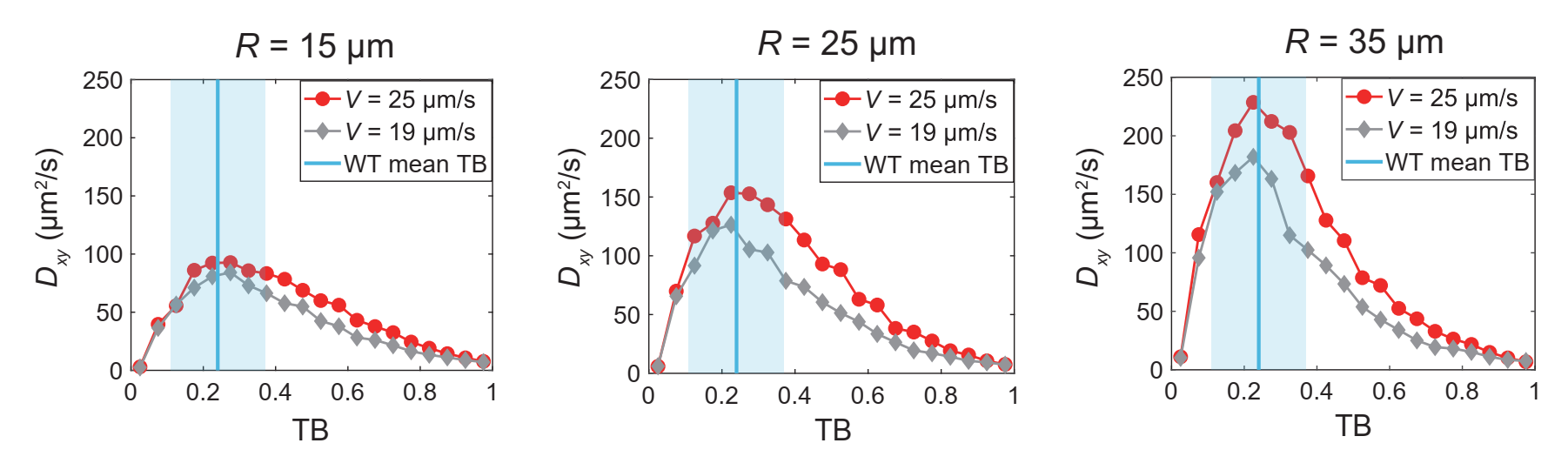


**Figure S7.** Simulated relationship between *D_xy_* and TB at various *V* and *R* values. The swimming velocity *V* was set to 19 or 25 μm/s, which are the typical values for *E. coli* in the oxygen-free trap motility buffer and commonly used oxygenated motility medium, respectively. Three different values (15/25/35 μm) were applied to the radius (*R*) of curvature of surface circular trajectories in consideration of the phenotypic variation of *E. coli* cells. The blue line and shaded area indicate the mean and standard deviation for the tumble bias of wild-type *E. coli* cells, respectively.


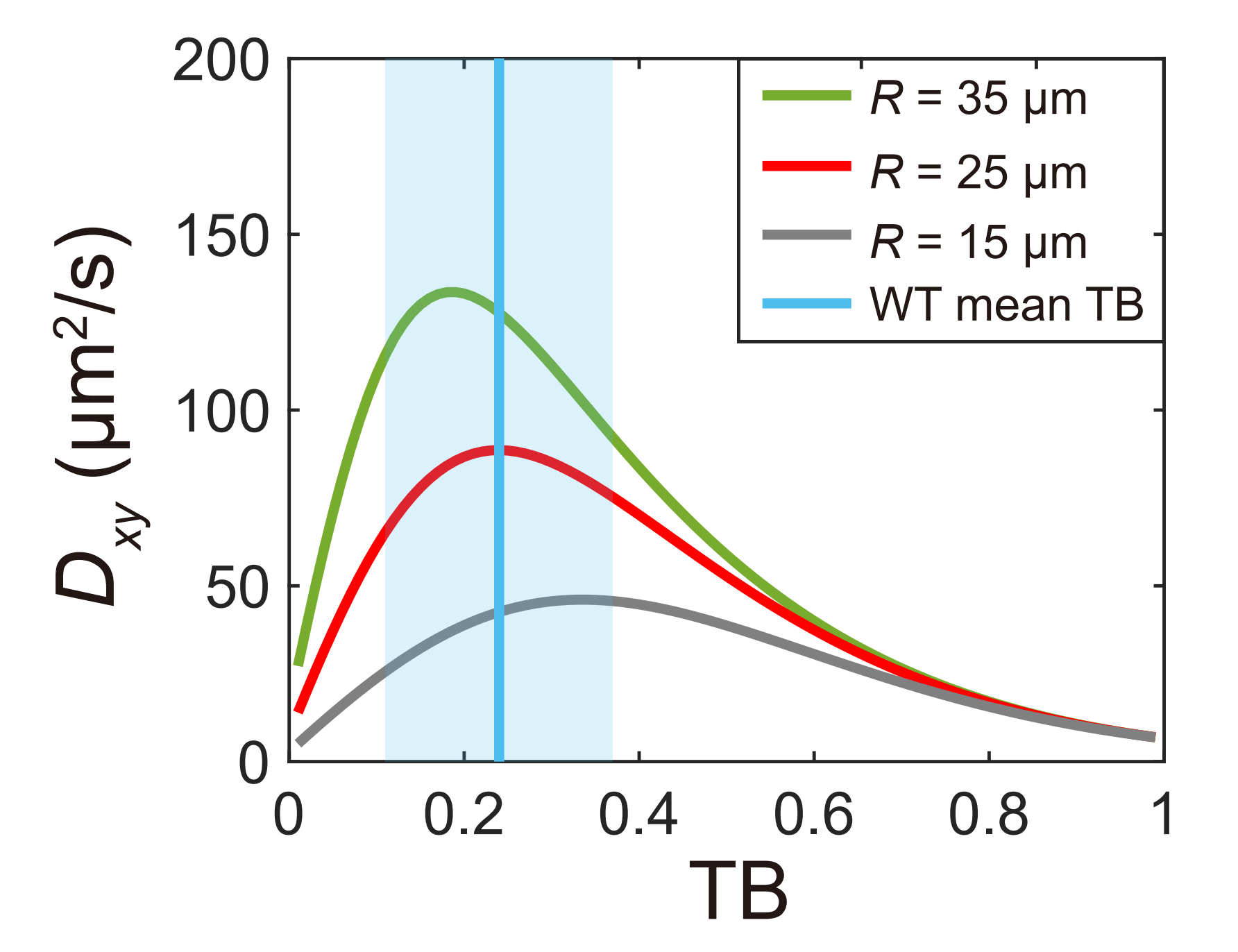


**Figure S8.** Direct computational relationship of *Dxy* versus TB by Equation (S20) without adjustment of *R*. The blue line and shaded area respectively indicate the mean and standard deviation for the tumble bias of wild-type *E. coli* cells.
